# Physicochemical, Microbial, and Nutritional Evaluation of Tiger Nut Yogurt as a Plant‐Based Alternative to Conventional Dairy Yogurt

**DOI:** 10.1002/fsn3.70897

**Published:** 2025-09-01

**Authors:** Regina Ofori Asante, Stephen Akese, Theophillus Mensah

**Affiliations:** ^1^ Department of Food and Postharvest Technology Koforidua Technical University Koforidua Ghana

**Keywords:** cow milk yogurt, microbial analysis, physicochemical properties, plant‐based alternative, tiger nut yogurt

## Abstract

The growing demand for plant‐based dairy alternatives, driven by increasing lactose intolerance and dairy allergies, has sparked interest in underutilized crops such as tiger nut ( *Cyperus esculentus* ). This study assessed the physicochemical, microbial, and compositional properties of tiger nut yogurt with (TMYS) and without (TMYNS) starter culture, compared to conventional cow milk yogurt (CMY). The CMY exhibited the highest pH (4.87), while TMYNS showed greater acidity (pH 4.42). Tiger nut yogurts had significantly higher fat content (1.79%–1.88%) compared to CMY (1.08%), indicating their enhanced nutritional value. Total soluble solids were also slightly lower in tiger nut yogurts. Although microbial loads were higher in TMYS and TMYNS (up to 1.1 × 10^5^ CFU/mL), *Escherichia coli* was detected only in CMY and not in any of the tiger nut formulations. Yeast and coliforms were present across samples, but no mold contamination was observed. These findings highlight the potential of tiger nut yogurt as a safe and nutritious non‐dairy alternative. The absence of *E. coli* in tiger nut‐based yogurts, despite higher total microbial loads, is noteworthy and suggests better microbial safety. This study contributes novel insight by evaluating tiger nut as a functional yogurt base using both inoculated and spontaneous fermentation methods. However, further research is recommended to improve sensory appeal and extend shelf stability through optimized processing and packaging.

## Graphical abstract

This study compared the physicochemical, microbial, and nutritional properties of tiger nut‐based yogurts (sweetened and unsweetened) with conventional dairy yogurt. Tiger nut yogurts exhibited comparable pH levels, titratable acidity (%), total soluble solids (°Brix), moisture (%), and microbial safety. These findings demonstrate the potential of tiger nut yogurt as a novel, plant‐based dairy alternative with nutritional and microbiological advantages.

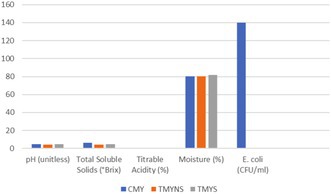

## 1 Introduction

Yogurt is a globally consumed fermented dairy product cherished for its nutritional composition, probiotic functionality, and appealing sensory characteristics (Pannerchelvan et al. 2024). Traditionally, it is made through the fermentation of cow milk by lactic acid bacteria, primarily *Lactobacillus bulgaricus* and *Streptococcus thermophilus* , which ferment lactose into lactic acid. This metabolic process not only imparts the distinctive tart flavor and creamy texture but also enhances the safety, shelf life, and digestibility of the product (Farag et al. 2022). Yogurt plays a crucial role in many diets due to its high‐quality protein content, calcium, B‐vitamins, and gut health‐promoting live cultures (Pannerchelvan et al. 2024). Despite its benefits, dairy yogurt presents dietary challenges for certain population groups. An increasing number of individuals are affected by lactose intolerance, cow milk protein allergies, and hypercholesterolemia (Pratelli et al. 2024). Moreover, plant‐based and vegan lifestyles are becoming more prevalent worldwide. Ethical concerns about animal welfare and the environmental footprint of dairy farming, including greenhouse gas emissions and high‐water usage, have driven consumer interest in plant‐based dairy alternatives (Rathod et al. 2025). In response, the food industry has developed non‐dairy yogurts derived from sources like soy, almond, oat, coconut, and rice. More recently, tiger nut ( *Cyperus esculentus* ) has emerged as a noteworthy candidate (Rathod et al. 2025; Sv et al. 2022).

Tiger nut is a nutrient‐dense, underutilized tuber grown widely in West Africa, Southern Europe, and parts of Asia. It has been traditionally used in the preparation of “horchata de chufa,” a non‐alcoholic beverage appreciated for its sweetness and refreshing nature (Vitali et al. 2023). Tiger nut's nutritional composition includes high levels of resistant starch, insoluble fiber, oleic acid, vitamins C and E, and essential minerals such as potassium, calcium, phosphorus, and magnesium (Ogunremi et al. 2024; Rebezov et al. 2023). It is naturally lactose‐ and gluten‐free, making it a suitable base for plant‐based milk and yogurt formulations, particularly for individuals with dietary restrictions. Its sweet taste reduces the need for added sugars, while its bioactive compounds, such as polyphenols and phytosterols, offer additional health benefits (Edo et al. 2023).

In recent years, the fermentation potential of tiger nut milk has attracted scholarly interest. Edo et al. (2024) demonstrated that tiger nut milk can support the proliferation of probiotic cultures and maintain suitable pH and titratable acidity profiles during fermentation. Vitali et al. (2023) reported that tiger nut‐based yogurts fermented with lactic acid bacteria retained good sensory acceptability and delivered viable probiotic counts. Other studies have explored its antioxidant capacity, anti‐inflammatory properties, and possible use in fermented symbiotic drinks and cheese analogues (Ogunremi et al. 2024). These studies indicate the growing potential of tiger nut in the functional food sector. However, most of these investigations were limited in scope, often focusing on a single aspect such as microbial viability, sensory analysis, or antioxidant capacity, rather than a comprehensive evaluation (Vitali et al. 2025).

Despite its promise, plant‐based yogurt faces several formulation challenges. The differences in protein and carbohydrate structures between plant‐based and dairy substrates affect fermentation kinetics, final acidity, and texture. For instance, tiger nut milk has a lower protein concentration and lacks casein, the major protein responsible for the gel network in dairy yogurt. These structural differences influence acid production, gel formation, viscosity, and water‐holding capacity properties critical to consumer acceptability (Grasso et al. 2020; Harper et al. 2022). Moreover, while plant‐based products are perceived as healthy, they can be susceptible to microbial contamination during preparation and storage, particularly in artisanal and small‐scale settings where hygienic standards and temperature controls may be suboptimal (Mahunu et al. 2024). Common spoilage organisms such as coliforms, *Escherichia coli* , yeasts, and molds can compromise safety and shelf life (Owusu‐Kwarteng et al. 2020).

A crucial but often overlooked factor in plant‐based yogurt formulation is the role of sugar. Sugar serves not only as a sweetener but also as a fermentation substrate for lactic acid bacteria. The presence or absence of added sugar may significantly influence microbial activity, acidification rate, and flavor profile (Sorensen 2024). While this has been well documented in dairy systems, few studies have explored the effect of sugar addition on the physicochemical and microbiological characteristics of tiger nut yogurt. Understanding these interactions is essential for optimizing product quality and fermentation efficiency.

In addition, accurate nutritional profiling, particularly fat and non‐fat content, is essential to determine the energy contribution and dietary suitability of the product (Cho et al. 2024). Consumers are increasingly health‐conscious and seek plant‐based products that are not only lactose‐free but also low in fat, high in fiber, and rich in functional nutrients. Therefore, analyzing fat and solid‐non‐fat content provides insights into the caloric value, texture, and nutritional appeal of the yogurt.

In the Ghanaian context, tiger nut is abundantly grown and locally appreciated, yet its industrial utilization remains limited (Novor and Donkor 2024). Promoting tiger nut yogurt production could diversify local food products, reduce dependency on imported dairy, and contribute to food security. Moreover, it provides opportunities for small‐scale processors, women entrepreneurs, and local supply chains. However, for tiger nut yogurt to gain market acceptance, it must meet safety, stability, and nutritional quality benchmarks comparable to established dairy yogurts.

This study was therefore designed to fill critical knowledge gaps by conducting a comprehensive evaluation of the physicochemical properties (pH, total soluble solids, titratable acidity), microbial quality (presence of coliforms, *E. coli* , yeasts, and molds), and proximate composition (fat and non‐fat solids) of tiger nut‐based yogurt with sugar (TMYS) and without sugar (TMYNS) compared to cow milk yogurt (CMY). The outcomes will provide evidence on the viability of tiger nut yogurt as a functional, safe, and nutritious alternative to conventional dairy yogurt. Findings from this work are expected to guide process optimization, product development, and informed consumer choices in the expanding plant‐based dairy sector.

## 2 Materials and Methods

The materials used in this study included various consumables, such as tiger nuts ( *Cyperus esculentus* ), cow milk, sugar, starter culture, strawberry flavor, and water. The tiger nuts, sugar, and flavoring agent were sourced from the Koforidua Central Market and stored at room temperature in the Food Product Development Laboratory of Koforidua Technical University until use. The starter culture was purchased from Micrite Supermarket in Accra. The study included three sample formulations: CMY, TMYS, and TMYNS. CMY was prepared using cow milk as the control, while both TMYS and TMYNS were formulated with tiger nut milk. However, TMYS contained added sugar, whereas TMYNS was sugar free due to the naturally high sugar content of tiger nuts. Fresh tiger nuts were cleaned by removing any foreign or damaged nuts that could affect the taste and quality of the milk. The nuts were then washed and rinsed with distilled water before being soaked overnight to soften the fibers. 500 g of tiger nuts was blended multiple times with 1 L of warm water. The resulting mash was strained through a sieve to extract the milk, followed by additional straining to achieve a smooth and delicate consistency. This sample (CMY) served as the control in comparing the physicochemical and microbial properties of the tiger nut yogurt formulations.

### 2.1 Preparation of Tiger Nut Yogurt

Fresh tiger nuts ( *Cyperus esculentus* ) were procured from Adweso Market in Koforidua, located in the Eastern Region of Ghana. Before processing, defective and foreign materials were manually removed to ensure the quality and sensory attributes of the final product. The tiger nuts were washed thoroughly and rinsed with distilled water, then soaked in water overnight at ambient temperature to soften the fibrous content. A portion of 500 g of soaked tiger nuts was blended with 1 L of warm water using a high‐speed blender. The resulting slurry was filtered through a fine mesh sieve to extract the milk and subsequently filtered again to enhance the smoothness and consistency of the extract. The extracted tiger nut milk was pasteurized at 90°C for 15 min and then allowed to cool to 43°C. A starter culture (0.10 g of crystalline form) was inoculated into the pasteurized milk and incubated at 43°C for 6 h, following the protocol described by Masiá et al. (2020). Post‐fermentation, 500 mL of the resulting yogurt was sweetened with sugar (0.14 kg) and flavored with strawberry, then dispensed into sterilized containers and labeled as TMYS (tiger nut yogurt sweetened). The remaining 500 mL was left unsweetened but flavored with strawberry, bottled, labeled as TMYNS (tiger nut yogurt non‐sweetened), and refrigerated to arrest further microbial activity and fermentation.

### 2.2 Preparation of Cow Milk Yogurt (Control Sample)

Cow milk yogurt was prepared following the method described by Li et al. (2025) A total of 100 g of cow milk was dissolved in 1 L of potable water. The reconstituted milk was then subjected to pretreatment, homogenization, and pasteurization by heating to 85°C. During the heating process, the milk was gently stirred to prevent scorching, charring at the bottom, and boiling over. Following pasteurization, the milk was rapidly cooled using a cold‐water bath to a temperature range of 42°C–44°C. Once cooled, 5 g of commercial yogurt starter culture was inoculated into the milk and thoroughly mixed under aseptic conditions. The inoculated mixture was incubated undisturbed at 42°C–44°C for 6 h to allow fermentation and the development of the desired consistency and acidity. After the incubation period, the yogurt was rapidly cooled to arrest further fermentation and acid development. The final product underwent post‐fermentation handling, including gentle mixing, packaging in sterilized containers, and refrigeration for storage at 4°C.

### 2.3 Fat and Non‐Fat Content Determination

#### 2.3.1 Fat Content Analysis

The fat content of the yogurt samples was determined as described by Przybylska and Kuczaj (2024). This method is based on acid hydrolysis followed by solvent extraction. Approximately 3 g of homogenized yogurt sample was weighed accurately into a clean, dry conical flask. To this, 10 mL of concentrated hydrochloric acid (HCl) was added to facilitate the breakdown of the protein matrix and release the bound fat. The mixture was heated gently in a water bath at 70°C–80°C for 30 min, with occasional swirling, until complete hydrolysis was achieved. After hydrolysis, the flask was cooled to room temperature. 25 mL of ethanol was added followed by 25 mL of diethyl ether, and the flask was shaken vigorously for 1 min. Then, 25 mL of petroleum ether was added, and the mixture was again shaken. The upper organic layer containing the dissolved fat was carefully decanted into a pre‐weighed clean dry evaporation dish. This extraction step was repeated three times to ensure complete fat recovery. The pooled ether extracts were evaporated to dryness in a steam bath, and the dish was then dried in an oven at 105°C for 1 h, cooled in a desiccator, and weighed. All measurements were conducted in triplicate (*n* = 3), and mean values ± standard deviations were recorded for statistical analysis.

The fat content was calculated using the following formula:

$$\mathrm{Fat}\;\text{content}\left(\%\right)=\frac{\text{Weight of}\;\mathrm{fat}\;\text{residue}\;}{\text{Weight of sample}\;\left(g\right)}\times 100$$

#### 2.3.2 Non‐Fat Content Analysis

The non‐fat content was estimated according to the method described in Pearson's Composition and Analysis of Foods (Tulashie et al. 2022). Non‐fat solids (total solids − fat) were calculated by first determining the total solids and then subtracting the fat content. Approximately 5 g of yogurt sample was placed into a pre‐weighed aluminum moisture dish and dried in a hot air oven at 105°C for 4 h to obtain the total solids. The dish was cooled in a desiccator and weighed. The moisture content was determined by the difference in weight before and after drying. The total solids content was calculated as:

$$\text{Total solids}\;\left(\%\right)=100-\text{Moisture content}\;\left(\%\right)$$

Once total solids and fat content were obtained, the non‐fat solids content was calculated as:

$$\text{Non}-\mathrm{fat}\;\text{solids}\;\left(\%\right)=\text{Total solids}\;\left(\%\right)-\mathrm{Fat}\;\text{content}\;\left(\%\right)$$

All measurements were performed in triplicate, and the mean values were recorded for statistical analysis.

### 2.4 pH Determination

The pH of the pudding samples was determined using a digital pH meter (Hanna Instruments, Model H1991002). Measurements were conducted following the standard procedure outlined by the Association of Official Analytical Chemists.

### 2.5 Determination of Total Soluble Solids (°Brix)

Total soluble solids (TSS) of the samples were measured using a digital refractometer (Model H196801) at ambient temperature.

### 2.6 Titratable Acidity

Titratable acidity was determined following the method described by the Association of Official Analytical Chemists (AOAC) (2023). A 10 mL aliquot of each pudding sample was transferred into a 125 mL conical flask, followed by the addition of 100 mL of distilled water. Three drops of phenolphthalein indicator were added, and the mixture was titrated against 0.1 N sodium hydroxide (NaOH) until a persistent pale pink endpoint was observed. The volume of NaOH used was recorded, and titratable acidity was calculated as a percentage of citric acid, based on the method outlined by Walsh (2022). %TA = mL NaOH × Normality (NaOH) × 0.064 × 100.

$$\begin{array}{c} \text{Titrable acidity}\;\left(\%\right)\\ \;=\frac{\left(\text{Volume of NaOH}\times \text{Normality of NaOH}\times 0.064\times 100\right)\;}{\text{Volume of sample}\;\left(\mathrm{mL}\right)} \end{array}$$

where 0.064* = acid milliequivalent factor.

### 2.7 Moisture Content Determination

Moisture content was determined according to the method described by Mauer (2024). Aluminum moisture cans were first washed, oven‐dried at 103°C for 20 min, and then cooled in a desiccator. A 4 g portion of each sample was weighed into the pre‐weighed moisture can and placed in a hot air oven maintained at 103°C ± 2°C for 4 h. After drying, the sample was removed, cooled in a desiccator to prevent moisture absorption, and reweighed. Each sample was analyzed in triplicate to ensure reproducibility, and variability was managed through statistical computation of mean and standard deviation. The moisture content (%) was calculated using the following formula:

$$\text{Moisture content}\;\left(\%\right)=\frac{\text{Initial mass}-\text{Final mass}}{\text{Initial mass}}\times 100$$

### 2.8 Microbial Analysis

#### 2.8.1 Enumeration of Yeasts

Yeasts were enumerated using the pour plate method according to ISO 21527‐1:2008. Dichloran Rose Bengal Chloramphenicol (DRBC) agar (Oxoid CM; Oxoid Ltd., Basingstoke, Hampshire, UK) was used as the selective medium, with the addition of 1% chloramphenicol to inhibit bacterial growth. The medium was adjusted to pH 6.5. Plates were incubated uninverted at 25°C for 120 h, after which yeast colonies were counted. Microbial counts were performed in triplicate for each yogurt sample to ensure accuracy and consistency of the results.

#### 2.8.2 Enumeration of Aerobic Mesophilic Bacteria

Aerobic mesophiles were enumerated following the Nordic Committee on Food Analysis (NMKL No. 86, 2006). The pour plate method was employed using Plate Count Agar (PCA; Oxoid CM 325). Plates were incubated in the inverted position at 30°C for 72 h, and colony‐forming units (CFU) were recorded.

#### 2.8.3 Enumeration and Confirmation of Coliform Bacteria

Coliforms were quantified using the pour plate method following Wakabayashi et al. (2024). Two media were employed: Primary medium: Tryptone Tergitol Agar (TTA; Oxoid CM131, pH 7.3), used to resuscitate stressed microorganisms by incubation at room temperature for 2 h. Overlay medium: Violet Red Bile Lactose Agar (VRBA; Oxoid CM107, pH 7.4), applied as an overlay and incubated inverted at 37°C for 24 h. Suspected coliform colonies (reddish‐pink, ~0.5 mm in diameter) were confirmed by sub‐culturing into Brilliant Green Bile Broth (BGBB; Oxoid CM31) containing an inverted Durham tube. Tubes were incubated at 37°C for 24 h, and gas production was used as confirmation of coliform presence.

## 3 Results

### 3.1 Physicochemical Properties of Different Yogurt Samples Produced

The physicochemical properties of the different yogurt samples (CMY, TMYNS, and TMYS) varied significantly as shown in Table 1. The pH values ranged from 4.42 to 4.87, with CMY (cow milk yogurt) having the highest pH (4.87) and TMYNS (tiger nut yogurt without sugar) having the lowest (4.42). TMYS (tiger nut yogurt with sugar) had an intermediate pH (4.67). CMY had the highest TSS value (6.42°Brix), followed by TMYS (5.00°Brix), while TMYNS had the lowest (4.04°Brix). The presence of added sugar in TMYS increased its TSS compared to TMYNS, which lacked added sugar. The highest acidity was recorded in CMY (0.82%), followed by TMYS (0.71%), and the lowest was in TMYNS (0.62%). The addition of sugar in TMYS may have slightly promoted acid production by providing fermentable sugars for bacterial metabolism. All samples had relatively high moisture content, ranging from 80.30% to 82.00%, with TMYS having the highest value (82.00%).

**TABLE 1:** Physicochemical properties of different yogurt samples produced.

Parameter/samples	pH (unitless)	Total soluble solids (°Brix)	Titrable acidity (%)	Moisture (%)
CMY	4.87 ± 0.00ᵇ	6.42 ± 0.01^a^	0.82 ± 0.00^a^	80.30 ± 0.00^a^
TMYNS	4.42 ± 0.01^c^	4.04 ± 0.05^c^	0.62 ± 0.01^c^	80.42 ± 0.01^a^
TMYS	4.67 ± 0.01^a^	5.00 ± 0.00^b^	0.71 ± 0.01^b^	82.00 ± 0.00^a^

*Note:* Values are means ± standard deviations of the parameters; values with different superscripts within the same row are significantly different (*p* < 0.05).

Abbreviations: CMY, cow milk yogurt; TMYN, tiger nut milk yogurt sugar free; TMYS, tiger nut milk yogurt with sugar.

The fat and non‐fat content of the different yogurt samples (CMY, TMYNS, and TMYS) showed variations, reflecting the compositional differences between cow milk and tiger nut milk, as well as the influence of sugar addition (Table 2). The non‐fat content ranged from 10.11% to 10.96%, with CMY (cow milk yogurt) having the highest value (10.96%) and TMYS (tiger nut yogurt with sugar) having the lowest (10.11%). TMYNS (tiger nut yogurt without sugar) had a slightly higher non‐fat content (10.82%) compared to TMYS. The fat content varied significantly across the samples, with CMY having the lowest fat content (1.08%) and TMYS the highest (1.88%). TMYNS also had a relatively high fat content (1.79%).

**TABLE 2:** Fat and non‐fat content of different samples of yogurt produced.

Samples/parameters	Non‐fat (%)	Fat (%)
CMY	10.96 ± 0.00^b^	1.08 ± 0.02^a^
TMYNS	10.82 ± 0.00^a^	1.79 ± 0.02^b^
TMYS	10.11 ± 0.00^b^	1.88 ± 0.04^a^

*Note:* Values are means ± standard deviations of the triplicate determination; values with different superscripts within the same column are significantly different (*p* < 0.05).

Abbreviations: CMY, cow milk yogurt; TMYN, tiger nut milk yogurt sugar free; TMYS, tiger nut milk yogurt with sugar.

### 3.2 Microbial Analysis of Different Samples of Yogurt Produced

The microbial analysis revealed that tiger nut yogurts (TMYS and TMYNS) had a significantly higher aerobic plate count (1.1 × 10^5^ and 5.5 × 10^4^ CFU/mL, respectively) compared to cow milk yogurt (CMY) (1.9 × 10^3^ CFU/mL) as shown in Table 3. *E. coli* was detected only in CMY (1.4 × 10^2^ CFU/mL) but was absent in both TMYS and TMYNS. Coliforms were present in all samples, with CMY showing the highest count (2.6 × 10^2^ CFU/mL), followed by TMYS (1.63 × 10^2^ CFU/mL) and TMYNS (1.0 × 10^2^ CFU/mL). Yeast was also detected in all yogurts, with CMY having the highest count (6.0 × 10^2^ CFU/mL), followed by TMYS (5.4 × 10^2^ CFU/mL) and TMYNS (2.0 × 10^2^ CFU/mL). No mold was found in any of the samples, indicating the absence of fungal contamination.

**TABLE 3:** Microbial analysis of different samples of yogurt produced (CFU/mL).

Sample	APC (CFU/mL)	*E. coli* (CFU/mL)	Coliform (CFU/mL)	Yeast (CFU/mL)	Mold (CFU/mL)
CMY	1.9 × 10^3^	1.4 × 10^2^	2.6 × 10^2^	6.0 × 10^2^	NF
TMYS	1.1 × 10^5^	NF	1.63 × 10^2^	5.4 × 10^2^	NF
TMYNS	5.5 × 10^4^	NF	1.0 × 10^2^	2.0 × 10^2^	NF

*Note:* Values are means of triplicate determinations.

Abbreviations: APC, aerobic plate count; NF, not found.

## 4 Discussion

The study successfully evaluated and compared the physicochemical, microbial, and nutritional characteristics of tiger nut‐based yogurts (with and without sugar) and conventional cow milk yogurt. The results showed that tiger nut yogurts had higher fat content and comparable sensory attributes, which may have enhanced consumer acceptability due to their creamy texture. In terms of microbial quality, tiger nut yogurts exhibited higher aerobic plate counts but did not show detectable *E. coli* , unlike cow milk yogurt, which had a lower overall microbial load but tested positive for *E. coli* , indicating possible post‐pasteurization contamination. Coliforms and yeasts were found in all samples, indicating the need for improved sanitation and storage practices. No mold was detected in any sample, indicating that fermentation and storage conditions were well managed. The findings met the study's objective by providing a comprehensive comparison of the key quality and safety parameters of tiger nut yogurt and cow milk yogurt and confirmed the potential of tiger nut yogurt as a viable plant‐based dairy alternative.

### 4.1 Physicochemical Properties

The physicochemical properties of the yogurts in this study showed clear differences between cow milk yogurt (CMY) and the tiger nut‐based yogurts (TMYNS and TMYS), as well as the effect of sugar addition. These variations were mainly due to compositional differences between cow milk and tiger nut milk, as well as the fermentation process.

The pH values ranged from 4.42 to 4.87, with CMY recording the highest pH (4.87) and TMYNS the lowest (4.42). These results aligned with Küçükgöz et al. (2024), who reported increased acidification in non‐dairy substrates during fermentation. Similarly, Harper et al. (2022) noted that plant‐based milks supported different microbial activity, which affected acid production. The lower pH observed in tiger nut yogurts indicated higher acid production, consistent with studies showing that plant‐based yogurts tend to be more acidic due to differences in carbohydrate composition and fermentation behavior. TMYS had a lower pH than TMYNS, suggesting that sugar addition promoted acid formation by supplying more fermentable sugars to the lactic acid bacteria. This trend was also observed in soy‐based yogurts (Zang et al. 2024).

The total soluble solids (TSS) content was highest in CMY (6.42°Brix), followed by TMYS (5.00°Brix) and TMYNS (4.04°Brix). The increase in TSS in TMYS compared to TMYNS reflected the influence of added sugar, as also reported by Zang et al. (2024), who found higher TSS in sweetened plant‐based yogurts. The higher TSS in CMY was likely due to its naturally higher content of proteins and fats, as previously observed in dairy‐based yogurts (Fructuoso et al. 2021). Jiang et al. (2025) similarly noted that sugar addition contributed significantly to TSS values in plant‐based yogurts.

Titratable acidity was highest in CMY (0.82%), followed by TMYS (0.71%) and TMYNS (0.62%). The higher acidity in CMY supported findings by Nelios et al. (2023), who reported that cow milk yogurt tends to develop more lactic acid due to its higher lactose and protein content. The slightly lower acidity in TMYS and TMYNS could be linked to differences in sugar type and content in tiger nut milk, which may have influenced bacterial metabolism during fermentation. Aydar et al. (2021) also reported that plant‐based yogurts often showed lower acidity than dairy yogurts.

Moisture content ranged from 80.30% to 82.00%, with TMYS showing the highest value. All samples had high moisture levels, which are essential for a desirable yogurt texture and mouthfeel. The addition of sugar may have improved water retention, consistent with Jiang et al. (2025), who noted similar effects in plant‐based yogurts. Differences among samples were not statistically significant, indicating that the type of milk (cow or tiger nut) had a limited effect on moisture content. These findings supported earlier work by Fructuoso et al. (2021), which reported comparable moisture content in both dairy and non‐dairy yogurts. The physicochemical properties observed in this study were in line with previous reports on plant‐based yogurts. Wang et al. (2025) and Aydar et al. (2021) confirmed that plant‐based yogurts generally had lower pH, acidity, and TSS compared to cow milk yogurt. Jiang et al. (2025) emphasized the influence of sugar addition on improving yogurt quality, as reflected in the increased acidity and TSS observed in TMYS. Similarly, Taye et al. (2021) attributed the higher acidity in cow milk yogurt to its natural lactose content, which serves as a key fermentable sugar for lactic acid bacteria.

### 4.2 Fat

The fat and non‐fat content of the yogurt samples (CMY, TMYNS, and TMYS) revealed key differences between cow milk and tiger nut milk, as well as the effect of sugar addition on final product composition. Tiger nut‐based yogurts (TMYNS and TMYS) contained significantly more fat than cow milk yogurt (CMY), while CMY had the highest non‐fat content. The fat content ranged from 1.08% in CMY to 1.88% in TMYS. The higher fat levels in tiger nut‐based yogurts align with previous studies, which show that tiger nut milk contains more lipids, especially monounsaturated fatty acids, than cow's milk, contributing to a richer, creamier texture (Irondi et al. 2025). In contrast, the lower fat content in CMY (1.08%) was expected, as cow milk typically contains less fat unless fortified with cream (Siegrist et al. 2024). The slight increase in fat content in TMYS (1.88%) compared to TMYNS (1.79%) suggested that sugar addition may have influenced fat retention. Jiang et al. (2025) reported similar observations in plant‐based yogurts, where sugar appeared to affect fat distribution. This effect may have resulted from interactions between sugar and fat molecules, which altered solubility and promoted greater fat retention during processing.

### 4.3 Non‐Fat Content

The non‐fat content was highest in CMY (10.96%) and lowest in TMYS (10.11%), with TMYNS showing an intermediate value of 10.82%. These differences were attributed to the natural fat composition of tiger nut milk and the absence of milk proteins like casein in plant‐based alternatives. The higher fat levels, particularly in TMYS, may have enhanced mouthfeel and creaminess qualities preferred by some consumers (Rune et al. 2025). However, from a nutritional standpoint, the increased fat content could have contributed to a higher caloric density, which might raise concerns for calorie‐conscious individuals (Jiang et al. 2025). The elevated non‐fat content in CMY was consistent with prior research showing that cow milk typically contained higher proportions of proteins and lactose, which increased the non‐fat solids in yogurt (Paszczyk and Tońska 2022). The reduced non‐fat content in TMYS compared to TMYNS suggested that sugar addition may have caused a dilution effect, lowering the relative concentration of non‐fat solids in the final product. This trend was also observed by Fructuoso et al. (2021), who reported similar effects in other plant‐based yogurts containing added sugar. These findings aligned with previous studies on the physicochemical properties of plant‐based yogurts. Irondi et al. (2025) found that tiger nut milk had higher fat content than cow milk, contributing to a richer yogurt texture. Similarly, Jiang et al. (2025) reported that plant‐based yogurts such as soy and tiger nut generally had higher fat content than dairy‐based yogurts. Paszczyk and Tońska (2022) also confirmed that cow milk yogurts tended to have higher non‐fat content due to their greater protein and lactose composition. Additionally, Rana et al. (2021) observed that sugar addition in plant‐based yogurts often reduced non‐fat solid content, a pattern also noted in TMYS.

### 4.4 Microbial Analysis

The microbial analysis of the yogurt samples provided insights into their quality, safety, and potential shelf life. Notable differences in microbial loads across the samples were likely due to variations in composition, processing methods, and hygiene practices.

The aerobic plate count (APC) was significantly higher in tiger nut yogurts TMYS (1.1 × 10^5^ CFU/mL) and TMYNS (5.5 × 10^4^ CFU/mL) than in cow milk yogurt (CMY: 1.9 × 10^3^ CFU/mL). However, all values remained within the acceptable limit of 1 × 10^6^ CFU/mL set by food safety authorities such as the FDA and EU Commission for fermented dairy products (El‐Sayed et al. 2022). These elevated counts may have resulted from the lack of rigorous pasteurization and the nutrient‐rich nature of tiger nut milk, which supports microbial growth (Edo et al. 2023). The absence of antimicrobial proteins like lactoferrin and lysozyme in plant‐based milk might have further contributed to microbial proliferation (Kyrylenko et al. 2023). In contrast, CMY likely benefited from heat treatments such as pasteurization and homogenization, which reduced microbial presence. The relatively high APCs in TMYS and TMYNS suggested the need for improved pasteurization, better hygienic practices, and optimized storage to enhance microbial stability, even though levels remained within safety limits.

Interestingly, *E. coli* was detected only in CMY (1.4 × 10^2^ CFU/mL) but was absent in TMYS and TMYNS. According to Codex and FDA guidelines, *E. coli* should be absent in 1 mL of yogurt (Elsherif et al. 2024), so the presence in CMY indicates microbial quality non‐compliance and possible post‐pasteurization contamination, likely from poor handling, water quality, or equipment. The absence of *E. coli* in tiger nut yogurts might be due to antimicrobial compounds in tiger nut extract, such as polyphenols and alkaloids (Ogbeide et al. 2025). This finding suggests that, when properly handled, tiger nut yogurt could offer a microbiologically safer alternative to cow milk yogurt.

Coliforms were present in all samples, with the highest count in CMY (2.6 × 10^2^ CFU/mL), followed by TMYS (1.63 × 10^2^ CFU/mL) and TMYNS (1.0 × 10^2^ CFU/mL). These values exceed the typical acceptable limit of ≤ 10^2^ CFU/mL for fermented products (Some et al. 2021), indicating potential hygiene lapses during processing or packaging. As coliforms are standard indicators of sanitation, their presence across all samples reinforces the need for enhanced sanitary protocols and strict adherence to Good Manufacturing Practices (Fructuoso et al. 2021). The elevated coliform and *E. coli* levels in CMY further highlight post‐fermentation contamination risks.

Yeasts were also detected in all samples, with CMY showing the highest count (6.0 × 10^2^ CFU/mL), followed by TMYS (5.4 × 10^2^ CFU/mL) and TMYNS (2.0 × 10^2^ CFU/mL). All counts were below the spoilage threshold of 1 × 10^3^ CFU/mL (Taormina 2021), suggesting that the products were microbiologically acceptable at the time of analysis. However, yeast growth, especially in sugar‐containing samples like TMYS, might reflect early spoilage activity, as some yeasts thrive on residual sugars. The lower yeast count in TMYNS suggested that sugar omission helped limit yeast proliferation. Although yeasts can contribute to flavor development, uncontrolled growth may reduce product shelf life (He et al. 2024; Alkalbani et al. 2022).

No molds were detected in any of the samples, indicating an absence of fungal contamination. This positive outcome suggested effective fermentation control and proper storage conditions. The lack of molds also reduced the risk of spoilage and mycotoxin production, supporting product safety (Pouris et al. 2024).

The findings of this study aligned with earlier research. Xie et al. (2023) observed higher microbial loads in plant‐based yogurts due to their nutrient‐rich matrices. Similarly, Ntuli et al. (2023) linked *E. coli* presence in dairy products to poor post‐pasteurization handling, consistent with the detection of *E. coli* in CMY. The presence of coliforms across all samples was also in line with prior reports on contamination risks in both dairy and plant‐based yogurts (Fructuoso et al. 2021).

## 5 Conclusions

This study highlights the potential of tiger nut yogurt as a novel, plant‐based alternative to traditional cow milk yogurt, offering distinct nutritional and microbiological benefits. It is one of the few investigations to systematically compare the physicochemical and microbial characteristics of both sweetened and unsweetened tiger nut yogurts with conventional dairy yogurt, thereby addressing a gap in research on underutilized plant‐based milk sources. Tiger nut yogurts showed lower pH and titratable acidity than cow milk yogurt, indicating different fermentation patterns influenced by plant‐derived carbohydrates. The addition of sugar promoted acid production and increased total soluble solids, supporting active fermentation. These yogurts also had significantly higher fat content, which may enhance creaminess, but lower non‐fat solids, likely due to the absence of dairy proteins. A notable finding was the absence of *Escherichia coli* in all tiger nut yogurt samples, in contrast to its presence in cow milk yogurt. This suggests a potential microbial safety advantage of tiger nut milk, possibly due to intrinsic antimicrobial compounds or better handling practices. However, elevated aerobic plate counts and the presence of coliforms and yeast across all samples underscore the need for stricter hygiene, processing, and storage measures, especially in plant‐based systems that lack some of the natural antimicrobial protections found in dairy.

Importantly, no mold was detected in any sample, suggesting that current packaging and storage methods were effective in preventing fungal contamination and preserving short‐term shelf life.

In conclusion, this study provides valuable insights into tiger nut yogurt as a functional, lactose‐free dairy substitute with promising nutritional and microbiological qualities. Further research should focus on refining its formulation, improving sensory attributes, and enhancing microbial stability to support commercialization and wider consumer acceptance.

## Author Contributions

**Regina Ofori Asante:** conceptualization (equal), investigation (equal), supervision (equal), validation (equal), visualization (equal), writing – review and editing (equal). **Stephen Akese:** data curation (equal), methodology (equal), writing – original draft (equal). **Theophillus Mensah:** data curation (equal), formal analysis (equal), methodology (equal), writing – original draft (equal).

## Ethics Statement

The authors have nothing to report.

## Conflicts of Interest

The authors declare no conflicts of interest.

## Data Availability

Data will be made available base on request.
